# Graphene Nanoplatelet-Modified Portland Cement: Surface Microhardness and In Vitro Fibroblast Metabolic Activity

**DOI:** 10.3390/dj14100626

**Published:** 2026-10-02

**Authors:** Mohammad R. Islam, Jhurgen Caszo, Khalid Al Razaiki, Khalid Al Khaldi, Ayida Al Wahaibi, Abubaker Qutieshat

**Affiliations:** 1 Division of Dentistry, Faculty of Health, University of Dundee, Park Place, Dundee DD1 4HR, UK; 2 Veterinary Department, Royal Oman Police, Mounted Police Headquarters, Muscat 118, Oman; 3 Research Unit, Oman Dental College, Muscat 116, Oman; 4 Restorative Dentistry, College of Dental Medicine, University of Sharjah, Sharjah P.O. Box 27272, United Arab Emirates

**Keywords:** graphene nanoplatelets, Portland cement, surface microhardness, MTT metabolic activity, calcium silicate cement

## Abstract

**Background**: White Portland cement (PC) is compositionally related to mineral trioxide aggregate (MTA) and provides an experimental platform for evaluating targeted modification of calcium silicate materials. This study investigated the effects of graphene nanoplatelet (GNP) incorporation on surface microhardness, cellular metabolic activity and mineral-related characteristics of PC. **Methods**: White PC was modified with 1%, 2% or 3 wt.% GNP. Surface microhardness was assessed on independently prepared discs, with three indentations averaged to obtain one specimen-level value. Day-14 MTT metabolic activity of WPS9 human periradicular fibroblasts was evaluated using direct-contact (*n* = 4 per group), filtered-eluate (*n* = 6 per group) and insert-based assays (*n* = 3 per group, except MTA, *n* = 2). Endpoint pH, cell morphology and association, vimentin-associated fluorescence, surface deposits and X-ray diffraction (XRD) profiles were assessed descriptively. **Results**: Surface microhardness differed significantly among the formulations (Welch’s F(3,14.15) = 190.36, *p* < 0.001), increasing from 44.9 ± 4.9 HV for PC to 142.2 ± 33.0, 177.5 ± 43.4 and 299.3 ± 33.1 HV for 1%, 2% and 3% GNP-PC, respectively. All GNP-containing formulations had significantly greater hardness than PC, while 3% GNP-PC was significantly harder than the 1% and 2% formulations. No significant group differences in day-14 MTT metabolic activity were detected in the direct-contact or insert-based assays. In the filtered-eluate assay, all GNP-containing formulations produced significantly higher metabolic activity than PC eluate (*p* < 0.001). Endpoint pH values were 13 for PC and 1% GNP-PC and 8 for the 2% and 3% formulations. Representative microscopy showed fibroblast-like morphology, cell association with the tested surfaces and vimentin-associated fluorescence. Descriptive XRD analysis of one specimen per formulation showed numerical differences in calcium phosphate-related signals, with 3% GNP-PC having the highest apatite-family classification. **Conclusions**: Under the tested conditions, GNP incorporation significantly increased PC surface microhardness, while filtered eluates from GNP-containing formulations produced higher day-14 MTT metabolic activity than PC eluate. The remaining outcomes provided descriptive formulation-related observations, and no formulation consistently outperformed the others across all measures. Further replicated mechanical, physicochemical and cellular evaluation is warranted.

## 1. Introduction

Preservation of dental pulp vitality remains a central objective of contemporary endodontic and restorative practice. When the pulp is exposed through caries, trauma or iatrogenic injury, the material placed in direct contact with the pulp wound plays a critical role in reparative dentine bridge formation, resolution of inflammation, and long-term tooth retention [1]. An ideal pulp-capping or root-end filling material should therefore be biocompatible, set predictably in the presence of moisture or blood, support mineralised tissue formation, possess sufficient mechanical stability after restoration, and provide a durable seal against bacterial ingress [1,2].

Calcium silicate-based cements, including mineral trioxide aggregate (MTA), are widely used in vital pulp therapy, apexification, perforation repair and root-end filling because of their biocompatibility, hydrophilic setting behaviour, sealing ability and capacity to support calcium phosphate deposition in biological fluids [2,3,4,5]. Despite these advantages, MTA has recognised limitations, including prolonged setting time, difficult handling, potential tooth discolouration and relatively high cost, which can restrict routine use in some clinical settings [3,5,6]. Newer calcium silicate-based materials, such as Biodentine, have been introduced to address some of these handling and setting limitations; however, continued investigation of foundational calcium silicate substrates remains relevant for isolating the effects of individual modifiers and informing the development of cost-effective and mechanically robust repair materials [6].

Portland cement (PC) has long served as an experimental platform for the development of calcium silicate-based dental materials. MTA itself emerged through refinement of a Portland cement-based formulation, including the incorporation of a radiopacifying component, and retains the same principal tricalcium and dicalcium silicate phases [4,6,7,8]. This material continuity makes white PC a rational base matrix for investigating targeted modifiers such as GNP. Commercial PC may nevertheless contain source-dependent trace elements and lacks a radiopacifier; consequently, translation into a dental formulation would require compositional control and further evaluation of radiopacity, handling, sealing, colour stability and nanoplatelet retention.

Targeted modification of calcium silicate-based cements is an established strategy for adjusting their physical, chemical and biological behaviour. Previous studies have shown that changes in cement composition, radiopacifier content, mixing liquid and powder-to-liquid ratio can influence clinically relevant properties, including setting behaviour, radiopacity, calcium ion release, pH, solubility and mechanical performance [9,10]. This supports the rationale for investigating additive-based modification as a way to improve the balance between mechanical reinforcement and biological compatibility in PC-based materials. However, improvements observed in structural or mechanical properties alone cannot establish biological safety or clinical suitability, and these domains require separate evaluation in dental models.

Graphene-based nanomaterials offer one possible route for reinforcing calcium silicate-based materials. Since the isolation of graphene by Novoselov and co-workers, graphene-family materials have attracted interest because of their high mechanical strength, large surface area, high aspect ratio, and capacity to modify the microstructure of composite systems [11,12]. Graphene nanoplatelets (GNP), consisting of short stacks of graphene sheets, are particularly attractive as practical additives because they are more scalable and cost-effective than monolayer graphene while retaining the planar, high-modulus character of the parent material [12,13]. Compared with more oxidised graphene derivatives, such as graphene oxide, GNP has a predominantly graphitic surface, which may influence cement hydration, dispersion behaviour, ion release and cell-material interactions differently. However, graphene-family nanomaterials can also produce concentration-, size-, surface-chemistry- and dispersion-dependent biological responses, making dose optimisation essential for materials intended to contact pulp or periradicular tissues [14,15]. The dark colour of GNP, an aesthetic limitation shared with MTA, may increase the potential for material or coronal discolouration and restrict its use in aesthetically sensitive areas [16]. In addition, whether nanoplatelets can be released from the hydrated cement matrix, and how any released material might interact with or distribute within surrounding tissues, remains to be established [16,17,18].

Evidence from cementitious and dental calcium silicate-based systems indicates that graphene-family additives can modify both material structure and biological response [16,17]. In structural cementitious systems, GNP incorporation has been reported to refine hydration products, reduce porosity and improve mechanical performance at low concentrations [13]; such evidence provides a mechanistic rationale for reinforcement but does not by itself establish biological safety or clinical suitability in dental applications. In dental calcium silicate-based systems, graphene nanosheets have been reported to enhance physicomechanical properties without compromising cytocompatibility [18]. In modified Angelus MTA, low concentrations of GNP increased carbon content and surface microhardness without altering the underlying crystal structure [19]. Previous work showed that graphene oxide nanoplatelet incorporation into PC improved surface microhardness while preserving fibroblast viability at low concentrations, although higher loading was associated with early cytotoxicity [20]. Graphene derivative, concentration and overall material formulation therefore emerge as important determinants of the mechanical and cellular behaviour of graphene-modified calcium silicate materials.

The present study focused on GNP powder as a reinforcing phase for white PC because its predominantly graphitic surface may provide a different balance between mechanical reinforcement and cellular response compared with more oxidised graphene derivatives. Although GNP has been investigated in structural cementitious systems and modified MTA, its concentration-related effects in PC have not been examined across surface microhardness, complementary in vitro cellular-exposure configurations, endpoint pH and surface mineral-related characteristics within a single experimental framework. Examining these outcomes together is relevant because the concentration producing the greatest mechanical reinforcement may not produce corresponding changes across cellular and physicochemical outcomes.

The aim of this study was to prepare GNP-modified white PC formulations and investigate the effects of different GNP concentrations on surface microhardness, WPS9 fibroblast metabolic activity, endpoint pH and qualitative cellular and surface mineral-related characteristics.

## 2. Materials and Methods

### 2.1. Cell Culture

Human peri-radicular fibroblasts (WPS9) were used to assess the cytocompatibility of the tested materials. The cells were originally obtained from an in-house explant culture established from the soft bony crypt tissue of a patient at the Dundee Dental School and Hospital. Cells were maintained in Minimum Essential Medium (MEM) supplemented with 10% heat-inactivated foetal calf serum, 1 mM sodium pyruvate, 2 mM L-glutamine, non-essential amino acids, and 100 U/mL penicillin–streptomycin. Cultures were incubated at 37 °C in a humidified atmosphere containing 5% CO_2_.

### 2.2. Material Preparation

Four Portland cement-based formulations were prepared by incorporating graphene nanoplatelet powder (GNP) (Graphitene, Flixborough, Scunthorpe, UK, #GN7.5-P-1) into white Portland cement (Blue Circle, product identifier 2345676003072, Birmingham, UK) at 0, 1, 2, and 3 wt.%. The experimental groups were designated as PC, PC + 1% GNP, PC + 2% GNP, and PC + 3% GNP. In this study, PC refers to the unmodified white Portland cement formulation used as the experimental base material, whereas MTA refers to ProRoot MTA (product code EVM015; supplied by Dental Directory Ltd., Witham, UK), which was used as a commercial calcium silicate-based comparator in selected assays. For each GNP-modified formulation, the weighed dry GNP and Portland cement powders were mixed in a sealed container using a vortex shaker for 60 s before deionised water addition.

For each formulation, 1.0 g of the blended powder, comprising PC and the specified GNP fraction, was mixed with 0.4 mL of sterile deionised water, giving a constant liquid-to-total-powder ratio of 0.4 mL/g. GNP was treated as part of the powder phase; therefore, maintaining the same liquid volume and total powder mass standardised the mixing conditions across all formulations and prevented water dosage from becoming an additional experimental variable [21].

The paste was placed into circular moulds measuring 10 mm in diameter and 2 mm in thickness. Specimens were allowed to set for 24 h in a humidified incubator maintained at 37 °C and 95% relative humidity. After setting, discs were removed from the moulds and sterilised by exposure to ultraviolet light for 5 min on each side using a CL-1000 Ultraviolet Crosslinker before use in subsequent experiments.

### 2.3. pH Assessment

To assess the alkalinising effect of the material formulations, ten discs from each group were placed in 20 mL of cell culture medium and incubated at 37 °C for two weeks. After incubation, the pH of the surrounding medium was measured using indicator strips. The pH assessment was used as a descriptive endpoint measure.

### 2.4. Surface Microhardness Testing

Surface microhardness was measured using a Through-Indenter Viewing (TIV) hardness tester (GE Measurement and Control, Groby, UK). Ten independently prepared discs were tested for each formulation. Valid indentation measurements were obtained from all PC, 1% GNP-PC and 2% GNP-PC discs (*n* = 10 per group). Two specimens in the 3% GNP-PC group did not yield valid indentation measurements, leaving eight specimens for analysis (*n* = 8). Specimens were tested on the as-cast surface that had been in contact with the mould, without grinding or polishing. Three indentations were made on each included disc. Before each measurement, the built-in TIV camera was used to select an intact, previously unindented area, thereby preventing overlap between indentations. The instrument settings were a 9.8 N load, 0 s dwell time, probe type 101, 5% diagonal-ratio setting and DIN 50150 conversion. The three measurements were averaged to produce one value per disc, and this disc-level mean was used as the experimental unit.

### 2.5. MTT Metabolic Activity Assays

Cellular MTT reduction activity was assessed as an indirect indicator of cellular metabolic activity. Because MTT absorbance reflects mitochondrial reductase activity, the assay was interpreted as a metabolic activity assay rather than as a direct measure of cell proliferation or cell number. Three complementary MTT-based approaches were used to examine the biological response of WPS9 cells to the tested materials.

At each assessment time point, the culture medium was removed by aspiration using a sterile plastic Pasteur pipette, and 1 mL of MTT solution, prepared at 1 mg/mL, was added to each well. Plates were incubated for 3 h at 37 °C. After incubation, the MTT solution was aspirated carefully to avoid disturbing the material discs or attached cells. The resulting formazan crystals were solubilised by adding 1 mL of dimethyl sulfoxide to each well, followed by agitation on a shaker for 20 min at room temperature. Absorbance was measured using a FLUOstar OPTIMA plate reader (BMG LABTECH, Ortenberg, Germany) at 550 nm, with a 620 nm reference wavelength. Reference-corrected values were calculated as OD550−OD620, after which the corresponding blank-well values were subtracted. These corrected OD values were used for statistical analysis.

For the direct-contact and insert-based assays, WPS9 cells (passage 12) were seeded at 5 × 10^5^ cells/mL. Each well received 500 µL of cell suspension, corresponding to 2.5 × 10^5^ cells per well. Cells maintained in complete MEM containing 10% FCS without a test material served as the untreated cell control.

For the reported day-14 analyses, each MTT assay configuration comprised a single experimental run. Each material observation was derived from a separately prepared disc and one corresponding well; assay-specific group and specimen numbers are provided below.

#### 2.5.1. Direct-Contact MTT Assay

Five analytical groups were evaluated: untreated cell control, PC, PC + 1% GNP, PC + 2% GNP, and PC + 3% GNP. Four independently prepared discs per material group were placed in separate wells, while the untreated control comprised four cell-containing wells without a material disc (*n* = 4 per group; total N = 20). Each disc/well contributed one optical-density value.

WPS9 cells were cultured in the presence of PC, PC + 1% GNP, PC + 2% GNP, and PC + 3% GNP discs in 24-well plates. Cells cultured in 10% MEM served as the untreated cell control. The material discs had been allowed to set for 24 h at 37 °C and approximately 95% relative humidity, followed by ultraviolet sterilisation as described above. No additional chemical preconditioning was performed before cell exposure. In this configuration, the cells were exposed to the material surface, soluble products released into the medium, and any particulate material released from the discs. Cell metabolic activity was evaluated on days 1, 7 and 14 using the MTT protocol described above.

#### 2.5.2. Filtered-Eluate MTT Assay

Five analytical groups were evaluated: untreated cell control, PC eluate, PC + 1% GNP eluate, PC + 2% GNP eluate, and PC + 3% GNP eluate. Six independently prepared discs per material group were eluted separately, and each disc-derived eluate was applied to one corresponding well. The untreated control comprised six wells without material eluate (*n* = 6 per group; total N = 30).

Material discs were immersed in culture medium for 24 h at 37 °C to generate material-conditioned eluates. The eluates were then filtered through a 0.2 μm syringe filter to remove particulate debris before being applied to WPS9 cells cultured in 24-well plates. This assay was designed to isolate the effect of soluble material products after removal of particulate debris and without direct contact between the cells and material discs. Cell metabolic activity was assessed on days 1, 7 and 14 using the MTT protocol described above.

#### 2.5.3. Insert-Based Indirect-Contact MTT Assay

Six analytical groups were evaluated: untreated cell control, PC, MTA, PC + 1% GNP, PC + 2% GNP, and PC + 3% GNP. Three independently prepared discs per material group were placed in separate inserts, while the untreated control comprised three cell-containing wells without a material disc (*n* = 3).

An insert-based indirect-contact assay was performed using BRAND Insert 2-in-1 inserts with a 0.4 μm PET membrane. This design allowed soluble material components to diffuse into the culture medium while preventing cement particles or debris from coming into direct contact with cells on the well floor. Inserts containing PC, MTA, PC + 1% GNP, PC + 2% GNP, or PC + 3% GNP discs were suspended above WPS9 cells in 24-well plates. Unlike the filtered-eluate assay, soluble products were released continuously from the material disc during the culture period rather than being generated during a separate 24 h elution step and transferred to the cells. Cell metabolic activity was assessed on days 1, 7 and 14 using the same MTT protocol.

A pilot material-interference assessment was also performed using cell-free material controls. For each GNP concentration, two cell-free wells were evaluated under each of the three assay configurations: direct contact, filtered eluate and insert based. These controls underwent the complete MTT and DMSO procedure but contained no WPS9 cells. The control wells were used only to assess material-associated assay interference and were not included in the statistical analyses.

### 2.6. Scanning Electron Microscopy

Scanning electron microscopy was used to examine both material surface morphology and WPS9 cell attachment to the tested discs. For cell attachment assessment, WPS9 cells were seeded onto material discs and cultured for 14 days. After incubation, specimens were fixed for 60 min at room temperature using 1.25% glutaraldehyde and 1% paraformaldehyde in 0.08 M cacodylate buffer containing 0.02% CaCl_2_. Samples were washed twice with cacodylate buffer and stored at 4 °C before post-fixation in 1% osmium tetroxide for 60 min.

Specimens were dehydrated through a graded ethanol series, washed in acetone, critical-point dried, mounted on aluminium stubs, sputter-coated with gold/palladium, and examined using a JEOL JSM-7400F scanning electron microscope (Akishima, Tokyo, Japan). Additional material discs were immersed in culture medium for 14 days and examined by SEM to assess surface deposits and crystal morphology after incubation.

### 2.7. Immunofluorescence Staining

Immunofluorescence staining was performed to assess vimentin expression in WPS9 cells following exposure to the tested materials. Cells were seeded into 24-well plates, and inserts containing the prepared material discs were placed into the wells. Cells cultured in 10% MEM alone served as the untreated cell control. After 14 days of incubation, cells were fixed using ice-cold methanol, washed with PBS, permeabilised with 0.2% Triton X-100, and blocked using goat serum in PBS containing Tween.

Cells were incubated overnight at 4 °C with rabbit anti-vimentin primary antibody (Cell Signaling Technology, Danvers, MA, USA, #5741). After washing, samples were incubated with an Alexa Fluor-tagged goat anti-rabbit secondary antibody (Cell Signaling Technology, USA, # 4412) in the dark. Nuclei were counterstained with DAPI. Fluorescent images were acquired using a CellSens Standard imaging system attached to an Olympus IX72 fluorescence microscope.

### 2.8. X-Ray Diffraction Analysis

Disc specimens of PC, PC + 1% GNP, PC + 2% GNP, PC + 3% GNP and MTA were immersed in DMEM at 37 °C for 14 days. Surface-associated material was then collected from one specimen per formulation for XRD analysis. Measurements were performed using a Shimadzu LabX XRD-6000 diffractometer (Shimadzu, Kyoto, Japan) with Cu radiation at 30 kV and 30 mA. Data were acquired in continuous *θ*-2θ mode over 5–100º 2*θ* at a scan speed of 2º/min; the exported profiles had an angular interval of 0.017°. The divergence and scatter slits were each 1º, and the receiving slit was 0.15 mm. No additional background subtraction or smoothing was applied to the displayed profiles. Relative peak areas assigned during the exploratory assessment to apatite-family and non-apatite CaP-related signal categories were tabulated descriptively.

### 2.9. Statistical Analysis

Quantitative data were analysed using R software (v 4.4.3). For surface microhardness, each independently prepared disc constituted the experimental unit. The mean of three indentations obtained from each disc was entered as one observation, and individual indentations were not treated as independent measurements. Disc-level distributions were assessed by inspection of Q-Q plots. Because group variances and sample sizes differed, microhardness was analysed using Welch’s one-way ANOVA followed by Games–Howell post hoc comparisons.

For the MTT assays, endpoint day-14 blank- and reference-corrected OD_550_-OD_620_ values were analysed separately for each assay configuration: direct-contact, filtered-eluate and insert-based assays. Each material observation represented one separately prepared disc and its corresponding well, while each untreated-control observation represented one cell-containing well. Technical measurements were not treated as additional independent observations. Residual distributions were assessed using Q-Q plots, and homogeneity of variance was evaluated using the Brown–Forsythe test. Differences between material groups were assessed using one-way analysis of variance followed by Tukey-adjusted post hoc comparisons. Effect sizes were reported as eta-squared. Although measurements were obtained on days 1, 7 and 14, formal between-group analysis and quantitative presentation were restricted to the prespecified day-14 endpoint. Days 1 and 7 were not included in inferential comparisons, and no group-by-time inference was made.

pH, SEM, immunofluorescence and XRD findings were analysed descriptively because these components were qualitative or based on representative or non-replicated observations. Statistical significance was set at *p* < 0.05.

## 3. Results

### 3.1. Surface Microhardness

Surface microhardness differed significantly among the formulations (Welch’s F(3,14.15) = 190.36, *p* < 0.001; Figure 1). Mean hardness values were 44.9 ± 4.9 HV for PC, 142.2 ± 33.0 HV for 1% GNP-PC, 177.5 ± 43.4 HV for 2% GNP-PC and 299.3 ± 33.1 HV for 3% GNP-PC. These represented increases of 216.7%, 295.3% and 566.5%, respectively, relative to PC. Games–Howell comparisons showed significantly greater hardness for all GNP-PC formulations compared with PC and for 3% GNP-PC compared with both 1% and 2% GNP-PC (all *p* < 0.001). The difference between 1% and 2% GNP-PC was not statistically significant (*p* = 0.211).

### 3.2. MTT Metabolic Activity and Cell Morphology

Representative phase-contrast images showed predominantly fibroblast-like cell morphology across the three MTT assay formats (Figure 2).

In the direct-contact assay, representative images showed visible particulate debris around unmodified Portland cement (PC), but debris release was not quantitatively assessed (Figure 2a). No significant differences in day-14 MTT metabolic activity were observed among the five groups, each represented by four independently prepared discs (F(4,15) = 0.82, *p* = 0.535, η^2^ = 0.18; Figure 3a). No Tukey-adjusted pairwise comparison among the untreated cell control, PC and the three GNP-modified formulations reached statistical significance.

In the filtered-eluate assay, a significant effect of material group was observed at day 14 among the five groups, each represented by six independently prepared disc eluates (F(4,25) = 35.03, *p* < 0.001, η^2^ = 0.85; Figure 3b). Mean blank- and reference-corrected OD_550_-OD_620_ values (mean ± SEM) were 1.047 ± 0.047 for the untreated cell control, 0.472 ± 0.035 for PC, 1.349 ± 0.062 for PC + 1% GNP, 1.224 ± 0.083 for PC + 2% GNP and 1.194 ± 0.054 for PC + 3% GNP. Tukey-adjusted post hoc comparisons showed that the untreated cell control and all three GNP-containing groups produced significantly higher MTT metabolic activity than PC (all *p* < 0.001). PC + 1% GNP was also significantly higher than the untreated cell control (*p* = 0.009). No significant differences were detected among the three GNP-containing groups.

In the insert-based assay, the analysis included three independently prepared discs per group, except for MTA, for which one well was excluded because it produced a blank-equivalent signal (*n* = 2; total N = 17). No significant differences in day-14 MTT metabolic activity were observed among the untreated cell control, PC, MTA and the three GNP-modified formulations (F(5,11) = 1.33, *p* = 0.320, η^2^ = 0.38; Figure 3c).

Cell-free material controls produced reference-corrected OD_550_-OD_620_ values ranging from 0.000 to 0.014 across the three GNP concentrations and assay configurations, consistent with negligible material-derived optical signal under the tested conditions.

### 3.3. pH Response

After two weeks of immersion in cell culture medium, all material groups yielded alkaline endpoint readings. PC and PC + 1% GNP produced the highest pH values, with both groups reaching pH 13. In contrast, PC + 2% GNP and PC + 3% GNP produced lower pH values, with both groups reaching pH 8.

### 3.4. Cell Attachment and Vimentin Expression

Representative SEM images acquired at ×1500 showed WPS9 fibroblasts associated with MTA, PC and the three GNP-modified PC surfaces after 14 days (Figure 4a). The images represent selected fields and were assessed qualitatively; cell coverage and morphology were not quantified, and no between-group morphometric comparisons were performed.

Representative immunofluorescence images showed green anti-vimentin-associated fluorescence and red-pseudocoloured DAPI-stained nuclei in the untreated cell reference and material-exposed conditions after 14 days (Figure 4b).

### 3.5. Mineral Precipitate Morphology

After 14 days of immersion in cell culture medium, surface-associated deposits were observed on all cement surfaces (Figure 5). In the representative fields shown, PC and PC + 2% GNP exhibited relatively uniform fine nodular deposits, with faceted polyhedral structures visible at higher magnification. PC + 1% GNP showed a more irregular surface morphology with larger near-spherical globular aggregates. PC + 3% GNP showed a continuous-appearing surface layer with stacked plate-like and angular features. MTA showed a heterogeneous distribution of coarse block-like aggregates. These descriptions relate to the selected representative fields; surface coverage and deposit composition were not quantified.

### 3.6. Descriptive XRD Assessment of Surface-Collected Material

X-ray diffraction (XRD) profiles were obtained from surface-collected material after 14 days of immersion (Figure 6A). One specimen from each formulation was examined. For visual presentation, each profile was normalised independently to its maximum intensity and vertically offset. The corresponding unnormalised profiles and original coordinate files are provided in Supplementary Figure S1 and Supplementary Data S1.

The descriptive relative peak-area classifications for apatite-family and non-apatite calcium phosphate (CaP)-related signals, respectively, were 7.2% and 32.1% for PC, 8.7% and 25.4% for PC + 1% GNP, 5.7% and 26.1% for PC + 2% GNP, 11.6% and 26.5% for PC + 3% GNP, and 6.1% and 28.4% for MTA (Figure 6B). Because one specimen per formulation was analysed and the phase-match scores were limited, these values were treated as descriptive classifications and were not used to estimate quantitative phase abundance, perform statistical comparisons or infer formulation-dependent mineral conversion or enrichment. Surface-collected material may also have included components of the underlying cement.

## 4. Discussion

The present study evaluated the effects of incorporating graphene nanoplatelets into Portland cement across mechanical, physicochemical and in vitro biological outcomes relevant to calcium silicate-based materials for vital pulp therapy. GNP incorporation markedly increased surface microhardness, with the highest value observed for PC + 3% GNP. The remaining outcomes varied according to formulation and assay configuration, suggesting that GNP loading influenced mechanical reinforcement, cellular metabolic activity, alkalinising behaviour and mineral-related responses differently rather than producing a uniform linear improvement across all outcomes.

The improvement in surface microhardness was the clearest material effect observed. This is consistent with the known reinforcing potential of graphene-family nanomaterials in cementitious systems, where their high aspect ratio and planar morphology have been associated with changes in mechanical behaviour and cement microstructure [22,23,24,25]. In the context of pulp-capping and endodontic repair materials, improved surface hardness may be clinically relevant because the material must resist disruption during placement of the overlying restoration and early function. However, mechanical reinforcement alone is insufficient for a material intended for vital pulp therapy, where direct or indirect interaction with inflamed or healing tissues is central to clinical performance.

The cytocompatibility findings are therefore important in defining the usefulness of GNP modification. Across the direct-contact and insert-based assays, GNP-modified PC did not show an overt reduction in WPS9 fibroblast metabolic activity compared with the control materials. This suggests that, under the tested conditions, GNP incorporation did not adversely affect WPS9 metabolic activity. This is clinically relevant because graphene-family materials can show concentration-dependent biological effects depending on particle size, surface chemistry, functionalisation, dispersion and exposure route [26]. The present findings also extend previous work on graphene oxide nanoplatelet-modified PC, where higher loading was associated with less favourable early cytocompatibility despite improved hardness [20]. Together, these observations support the view that graphene derivative type, concentration and surface chemistry are central determinants of the biological response to graphene-modified calcium silicate materials.

The different MTT assay designs also provide complementary information on the cellular response under different exposure conditions. Direct-contact testing exposed cells to the material surface, soluble products and any particulate material released from the cement. Filtered eluates emphasised the contribution of soluble components after removal of larger particulate debris, while the insert-based method allowed diffusion of soluble factors without direct particle contact. The stronger response observed in the filtered-eluate assay suggests that soluble products from GNP-modified PC may be more favourable to fibroblast metabolic activity once particulate material is removed. This response is therefore best interpreted as an improved MTT metabolic response to the soluble-material environment created by the GNP-modified formulations, rather than as direct evidence of proliferation alone. Because particle release was not quantified, this interpretation remains provisional and should be confirmed using particle-release analysis, inflammatory assays and additional cell-response endpoints.

Methodological variables are particularly important when evaluating calcium silicate-based materials in vitro [27,28,29]. Specimen geometry, surface area-to-volume ratio, setting conditions, elution time, extract dilution, medium composition and the selected cell model can all influence the measured biological response. Direct-contact assays expose cells to the material surface, soluble products and any released particles, whereas extract-based and insert-based models emphasise soluble components while reducing or preventing direct particle contact [27,28,29]. These differences help explain why biological responses may vary between assay configurations and support the use of complementary test designs rather than relying on a single cytocompatibility assay. In the present study, the filtered-eluate and insert-based assays were therefore interpreted alongside the direct-contact assay to explore how cellular responses varied when direct material and particulate contact was reduced or prevented. However, because MTT reduction reflects cellular metabolic activity, these findings should be interpreted as an indirect measure of cytocompatibility and should be complemented in future work by inflammatory mediator analysis, odontogenic differentiation markers, mineralisation assays and longer-term cell-response models.

The pH findings may help contextualise the different biological responses observed across the MTT assays. Calcium silicate-based cements release calcium and hydroxyl ions during hydration, and these ionic products contribute to alkalinity, apatite nucleation, mineralisation-related signalling, and hard-tissue formation [30]. At the same time, the biological response to calcium silicate materials is shaped by the exposure model, medium composition, extract conditions, specimen geometry, and the cellular endpoint being assessed [28]. In the present study, unmodified PC and PC + 1% GNP produced strongly alkaline conditions, whereas PC + 2% GNP and PC + 3% GNP produced a more moderate alkaline environment after two weeks of immersion. This interpretation is consistent with previous evidence that nano-modification of calcium silicate-based cements can influence physicochemical, mechanical and biological properties, including pH, calcium ion release, indentation hardness and biological response, although these effects depend on formulation and additive composition [31]. Similarly, particle-size modification of white MTA significantly increased calcium ion release and influenced pH elevation, supporting the concept that changes in cement particle/formulation characteristics can alter the soluble ionic environment of calcium silicate-based materials [32]. The lower pH observed with PC + 2% GNP and PC + 3% GNP may therefore reflect a formulation-dependent modification of hydration behaviour, ion diffusion or cement–medium interactions, although it should not be interpreted definitively as a GNP buffering effect because buffering capacity was not directly measured. The moderated pH response should also be interpreted as a potential biological trade-off rather than as an unequivocal advantage. While a less extreme alkaline environment may reduce early cellular stress and support fibroblast metabolic activity under the present in vitro conditions, high alkalinity is also one of the recognised contributors to the antibacterial behaviour of calcium silicate-based materials. Because antibacterial activity was not assessed in the present study, the lower pH observed in the 2% and 3% GNP groups should not be assumed to provide equivalent antimicrobial performance. The higher MTT metabolic activity observed with filtered eluates from the GNP-modified groups may be consistent with this interpretation, although the present data do not establish a direct causal relationship between pH and metabolic activity. Future studies measuring time-dependent pH, calcium ion release, antibacterial activity, and odontogenic differentiation markers in parallel would help define the precise physicochemical pathway involved.

SEM and immunofluorescence findings provided qualitative support for the cytocompatibility data by showing fibroblasts associated with the tested material surfaces and vimentin immunoreactivity across the exposure conditions. In the selected SEM fields, the more elongated cell morphology and denser filamentous extensions apparent on the higher GNP-containing formulations suggest that GNP may influence surface–cell interactions in a way that supports spreading and cytoskeletal organisation. This is consistent with reports that graphene-family materials can support cell adhesion, proliferation and differentiation in dental and bone-related cell models when used at appropriate concentrations [26,33,34,35,36]. Vimentin staining was used to visualise the fibroblastic cytoskeleton and was not interpreted as an odontogenic differentiation marker. Because the SEM and immunofluorescence assessments were qualitative, these observations should be interpreted as suggestive rather than definitive evidence of differences in cell behaviour. Quantitative morphometric analysis would be required to confirm differences in cell area, spreading, orientation or cytoskeletal organisation.

The mineral-related findings further suggest that GNP incorporation may modify surface precipitate formation on PC. The descriptive XRD relative peak-area analysis showed numerical differences in the calcium phosphate-related signal distribution, with PC + 3% GNP showing the highest apatite-family signal. This pattern may reflect formulation-dependent differences in precipitate formation, although the underlying mechanism was not directly tested. From a biological perspective, a greater apatite-family contribution is potentially relevant because apatite-like calcium phosphate is closer to the mineral phase of dental hard tissues than less mature calcium phosphate phases, such as octacalcium phosphate or related precursor phases [37,38]. Such an effect is plausible given evidence from cementitious materials showing that graphene derivatives can influence hydration products, microstructure and crystalline phase development [23,24,25,39]. Because the XRD findings were obtained from representative specimens, they should be interpreted as descriptive mineralisation trends that warrant confirmation in replicated phase and compositional studies. Similarly, the SEM-based differences in precipitate morphology remain descriptive because complementary compositional analysis, such as EDX or Raman spectroscopy, was not performed.

A key implication of the study is that no formulation consistently outperformed the others across all measured outcomes. PC + 3% GNP produced the highest surface microhardness and the highest numerical apatite-family classification, whereas no significant day-14 MTT differences were detected among the three GNP-containing formulations and PC + 2% GNP and PC + 3% GNP produced the same endpoint pH reading. This pattern suggests that the influence of GNP concentration is outcome-dependent rather than uniformly linear. For materials intended for vital pulp therapy, formulation development must consider mechanical reinforcement together with cellular response, alkalinising behaviour and surface mineral formation. The present findings therefore support further concentration optimisation rather than identifying a definitive optimal formulation. This is consistent with evidence from graphene-modified cementitious composites showing that excessive loading or inadequate dispersion may reduce performance through agglomeration or disruption of the cement matrix [25,39].

Several limitations should be considered. The cell-culture experiments used a single patient-derived periradicular fibroblast line, and MTT reduction provided a measure of metabolic activity rather than the broader cellular response. Further studies should include multiple donor-derived dental cell models, a positive cytotoxicity control and additional inflammatory, odontogenic and mineralisation-related endpoints. The small sample size of the insert-based assay also limited statistical precision. Material testing focused on surface microhardness, while other clinically relevant mechanical, handling and sealing properties remain to be investigated. GNP dispersion and agglomeration within the cement, together with possible concentration-dependent changes in water demand, rheology and handling at the fixed water-to-cement ratio, were not quantified. The pH assessment was limited to a descriptive two-week endpoint obtained using indicator strips. SEM observations were qualitative, while the XRD assessment was based on representative specimens; compositional analysis and replicated phase assessment would strengthen these findings. The possible effect of GNP incorporation on material colour also requires further investigation.

Beyond these experimental limitations, translation to a pulp-contacting material requires a broader, formulation-specific safety assessment. Studies of graphene-modified dental calcium-silicate cements indicate that physicochemical and biological responses vary with graphene form, concentration and cement matrix [16,18]. GNP release from hydrated GNP-PC was not quantified. Release of GNPs and other graphene-related materials has been reported from abraded epoxy composites, although these findings cannot be extrapolated directly to hydrated calcium-silicate cement [40]. Any released nano-objects would require characterisation of their release profile and toxicokinetics, including persistence, distribution and clearance [41]. Although the material-only blanks showed negligible absorbance under the present conditions, graphene can interact with tetrazolium reagents through adsorption, optical effects and electron transfer; confirmation using a colour-independent viability endpoint would therefore strengthen the cellular assessment [42]. Batch-specific elemental and leachate analyses are also needed because Portland cement may contain leachable trace metals, including arsenic, chromium and lead [43]. These considerations should be addressed through formulation-specific physicochemical and biological testing before the suitability of GNP-PC for pulp-contacting applications can be established.

Despite these limitations, the study provides a multimodal in vitro assessment of GNP-modified PC across mechanical, physicochemical and cellular-response domains. GNP incorporation markedly increased surface microhardness, while the MTT, pH, microscopy and XRD findings revealed formulation- and assay-dependent patterns. Collectively, the findings support continued optimisation of GNP-modified PC for potential use in vital pulp therapy and related endodontic repair applications, with future studies integrating replicated mechanical, physicochemical, cellular and mineral-related outcomes.

## 5. Conclusions

Under the tested conditions, incorporation of 1–3 wt.% GNP significantly increased the surface microhardness of PC, with PC + 3% GNP producing the highest mean value. In the filtered-eluate assay, the GNP-modified formulations produced significantly higher day-14 MTT metabolic activity than unmodified PC eluate, whereas no significant between-group differences were detected in the direct-contact or insert-based assays. The endpoint pH, microscopy and descriptive XRD findings provided complementary formulation-related observations, with PC + 3% GNP showing the highest numerical apatite-family signal. No formulation consistently outperformed the others across all measured outcomes. Collectively, these findings support continued optimisation of GNP-modified PC, with further replicated mechanical, physicochemical, cellular and mineral-related investigations required before its clinical relevance can be established.

## 6. Patents

The experimental GNP-PC formulation evaluated in this study is the subject of published international patent application PCT/OM2024/050004, publication number WO2026/038965, titled “Nanocarbon Platelets-Enhanced Portland Cement Composite”. All authors are listed as inventors on the application and therefore hold a potential intellectual-property interest in the tested formulation. No commercial product is currently marketed, and no licensing revenue or other financial benefit has been received from the application.

## Figures and Tables

**Figure 1:**
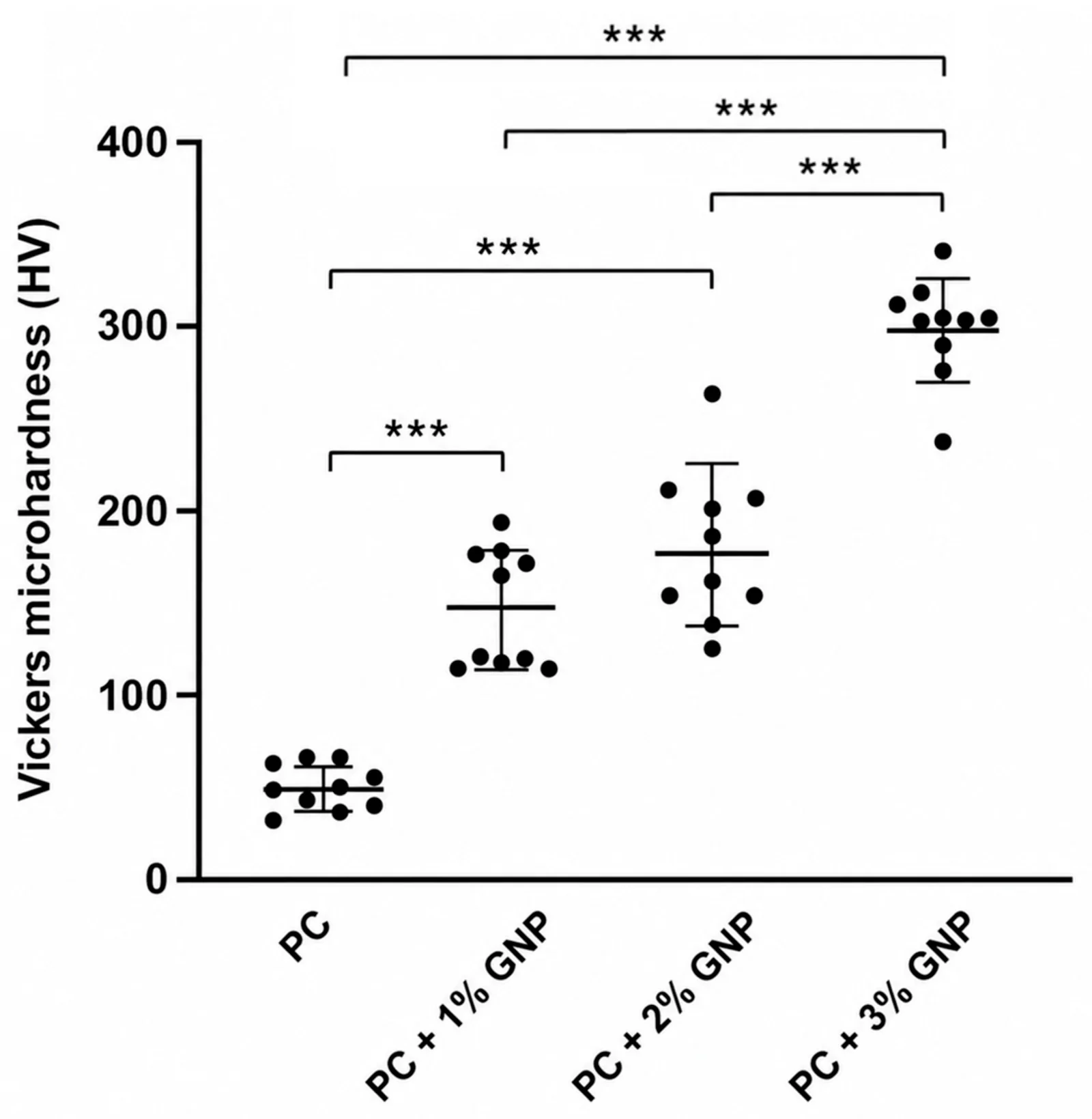
Surface microhardness of PC and GNP-PC formulations. Each point represents the mean of three indentations obtained from one independently prepared disc. Horizontal lines and error bars represent the mean ± SD. Sample sizes were *n* = 10 for PC, 1% GNP-PC and 2% GNP-PC, and *n* = 8 for 3% GNP-PC; ***: *p* < 0.001. GNP, graphene nanoplatelets; HV, Vickers hardness; PC, Portland cement.

**Figure 2:**
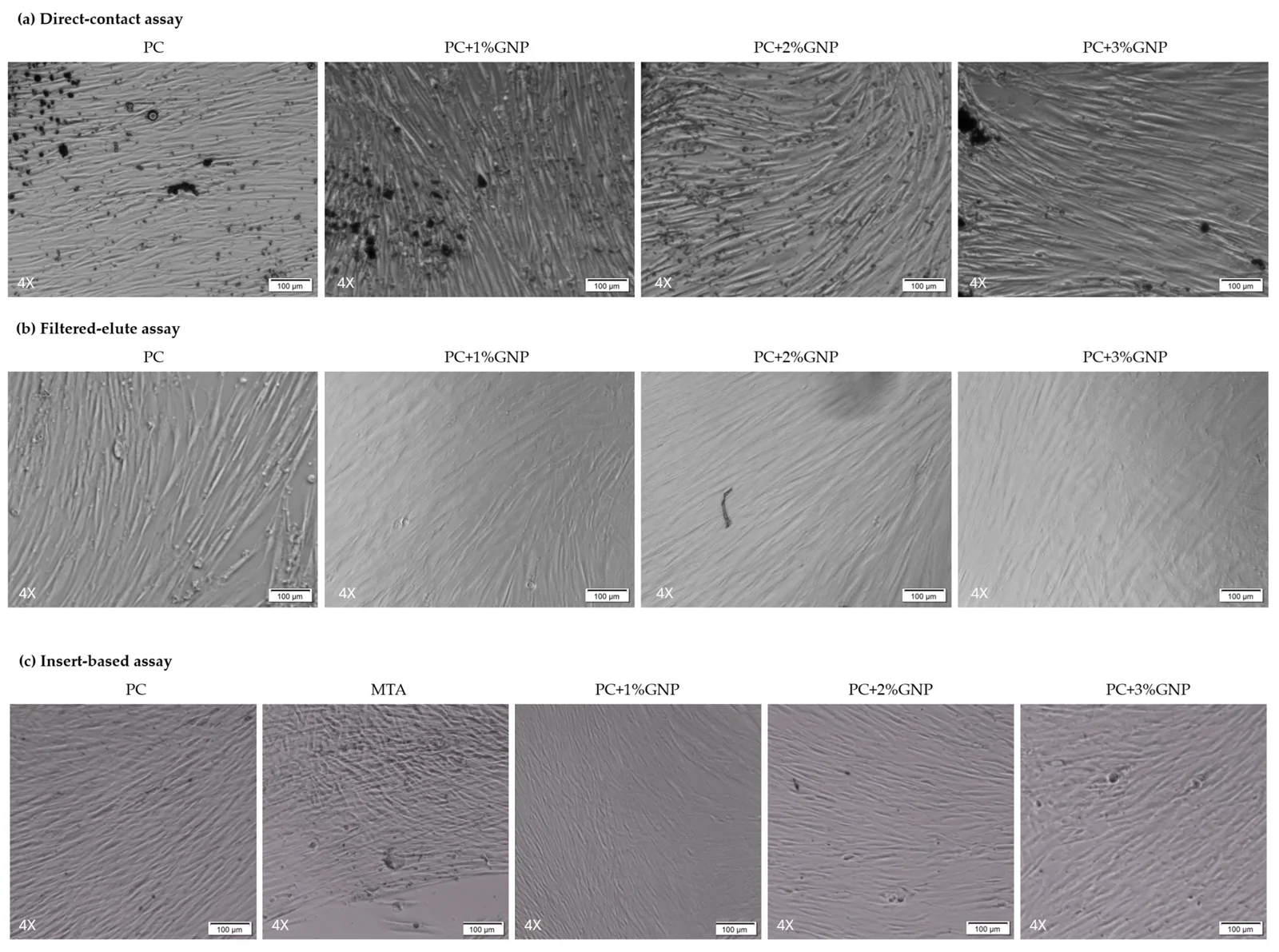
Representative phase-contrast microscopy images of WPS9 fibroblasts after 14 days of exposure to the tested materials. (**a**) Direct-contact assay. (**b**) Filtered-eluate assay. (**c**) Insert-based indirect-contact assay, including mineral trioxide aggregate (MTA) as a comparator. All images were acquired at 4× magnification; scale bars = 100 µm. Images are presented for qualitative morphological assessment only. GNP, graphene nanoplatelets; MTA, mineral trioxide aggregate; PC, Portland cement.

**Figure 3:**
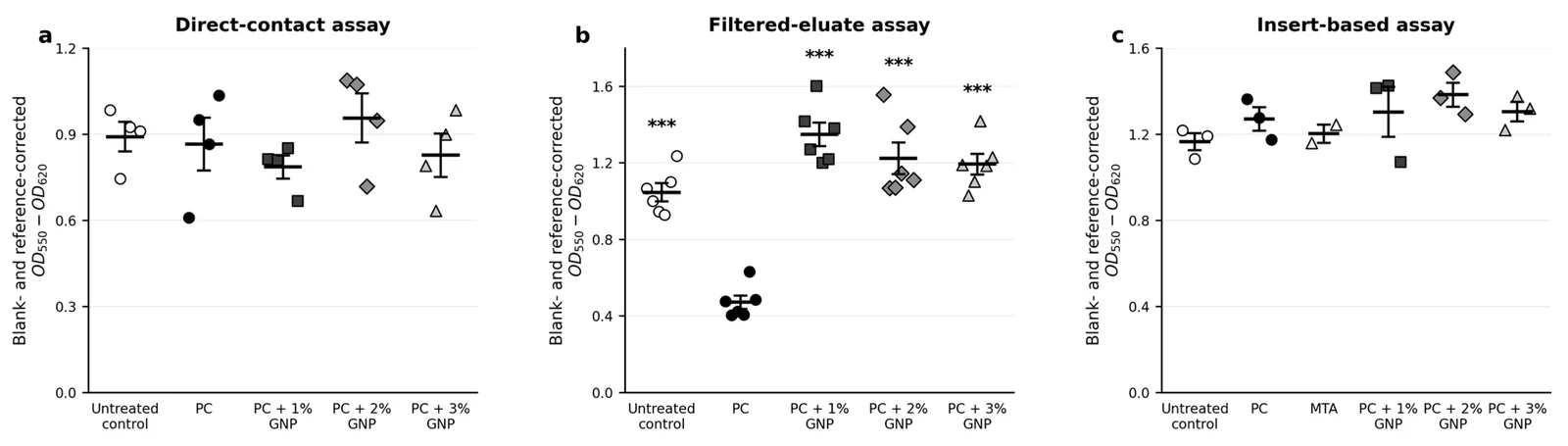
Day-14 MTT metabolic activity of WPS9 fibroblasts in the (**a**) direct-contact, (**b**) filtered-eluate and (**c**) insert-based assays. Each point represents one independent disc-associated assay observation or untreated cell-control well; horizontal bars and error bars indicate mean ± SEM. Values are presented as blank- and reference-corrected OD_550_-OD_620_ measurements. In the filtered-eluate assay, groups marked *** differed significantly from unmodified PC (*p* < 0.001; Tukey-adjusted comparisons). No significant pairwise differences were detected in the direct-contact or insert-based assays. GNP, graphene nanoplatelets; MTA, mineral trioxide aggregate; PC, Portland cement.

**Figure 4:**
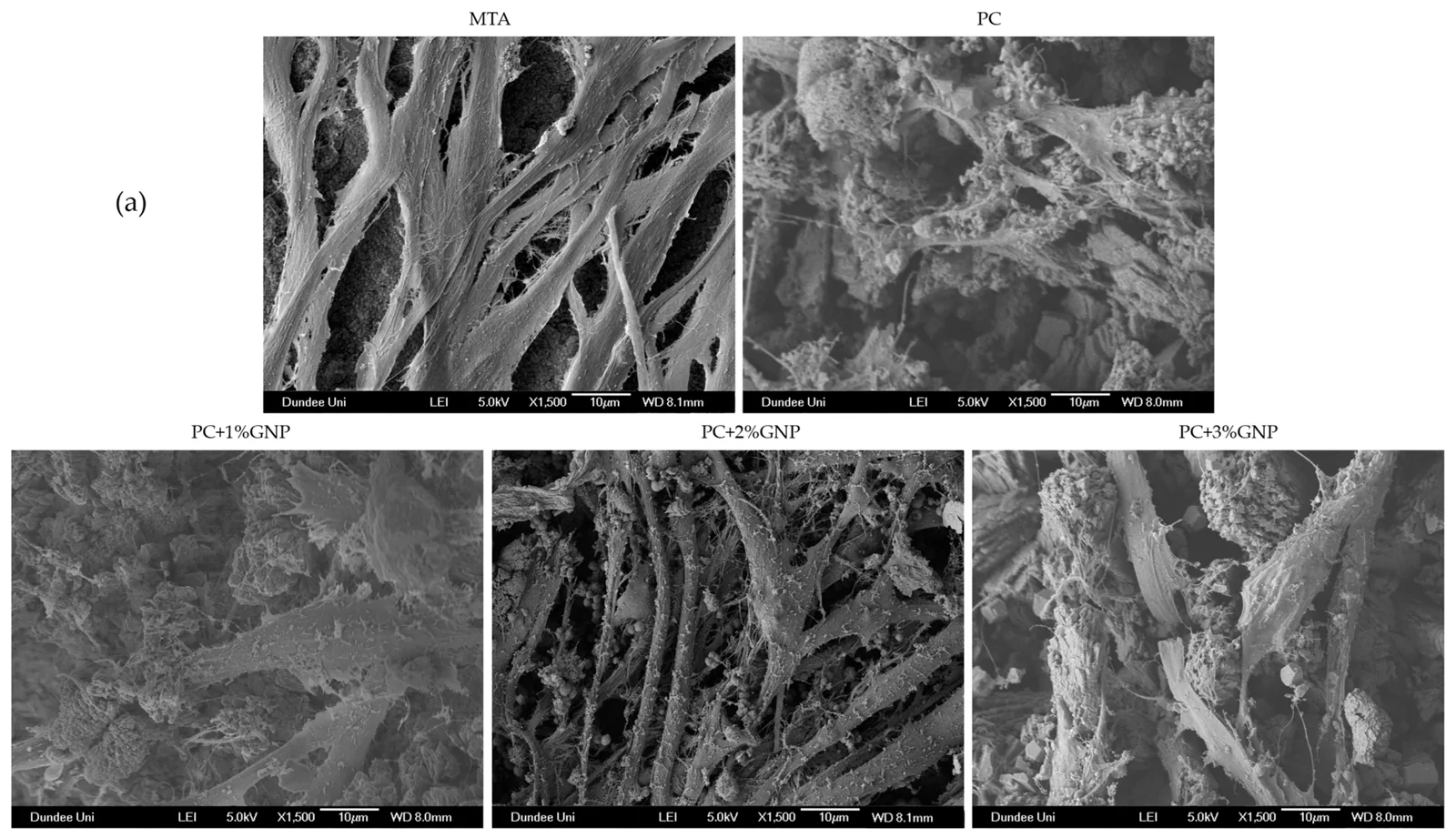

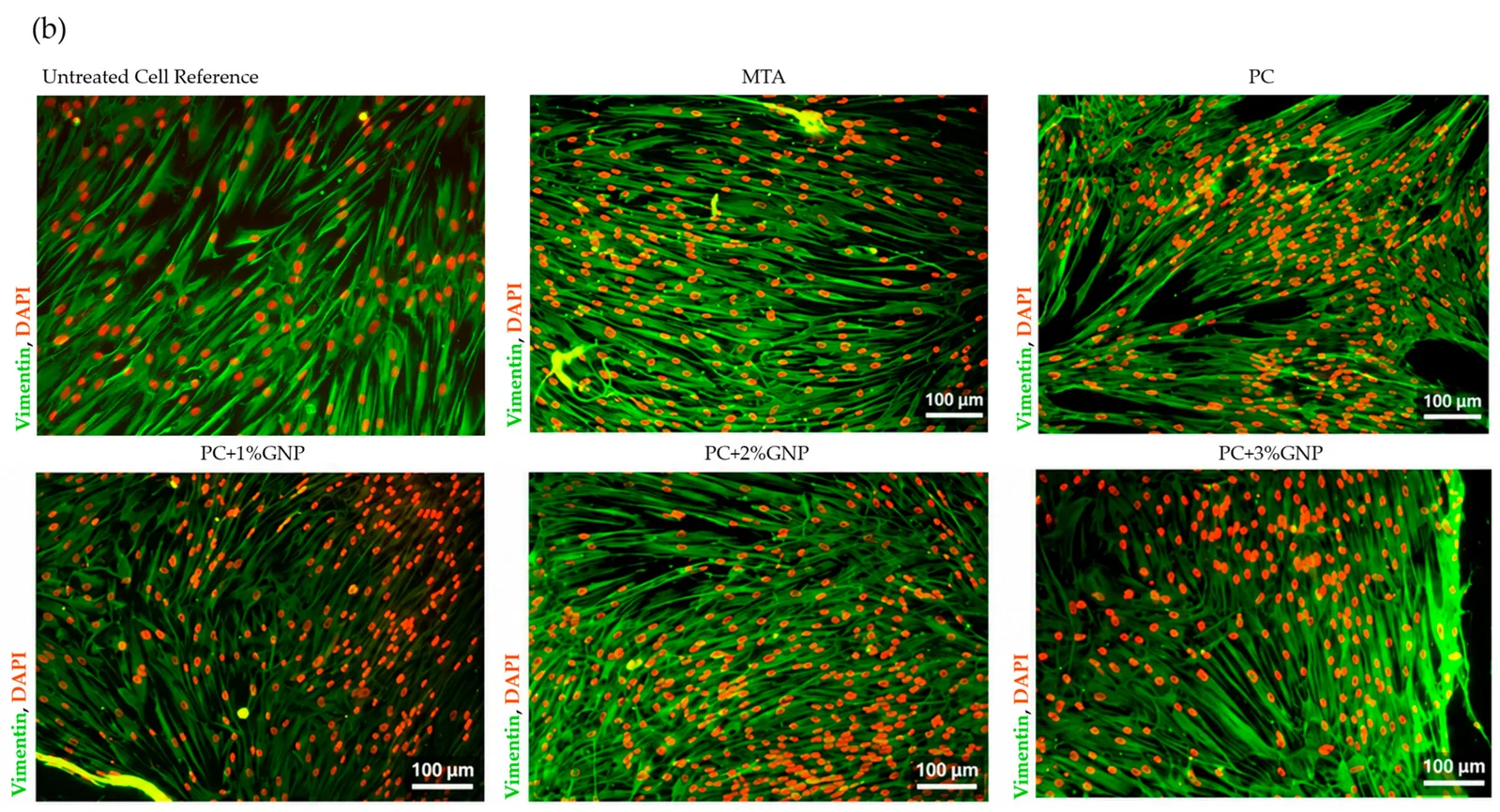
Representative scanning electron microscopy (SEM) and immunofluorescence images obtained after 14 days. (**a**) SEM images of WPS9 fibroblasts associated with mineral trioxide aggregate (MTA), unmodified Portland cement (PC), and PC containing 1%, 2%, or 3% graphene nanoplatelets (GNP). All SEM images were acquired at ×1500 magnification; scale bars = 10 µm. (**b**) Immunofluorescence images of an untreated WPS9 cell reference and WPS9 fibroblasts following insert-based indirect exposure to the corresponding materials. All displayed images were stained using a 1:100 dilution of the primary anti-vimentin antibody and acquired at 10× magnification; scale bars = 100 µm. Vimentin is displayed in green, and DAPI-stained nuclei are displayed in red pseudocolour. Images are presented for qualitative assessment only, and fluorescence intensity was not quantified. DAPI, 4′,6-diamidino-2-phenylindole; GNP, graphene nanoplatelets; MTA, mineral trioxide aggregate; PC, Portland cement; SEM, scanning electron microscopy.

**Figure 5:**
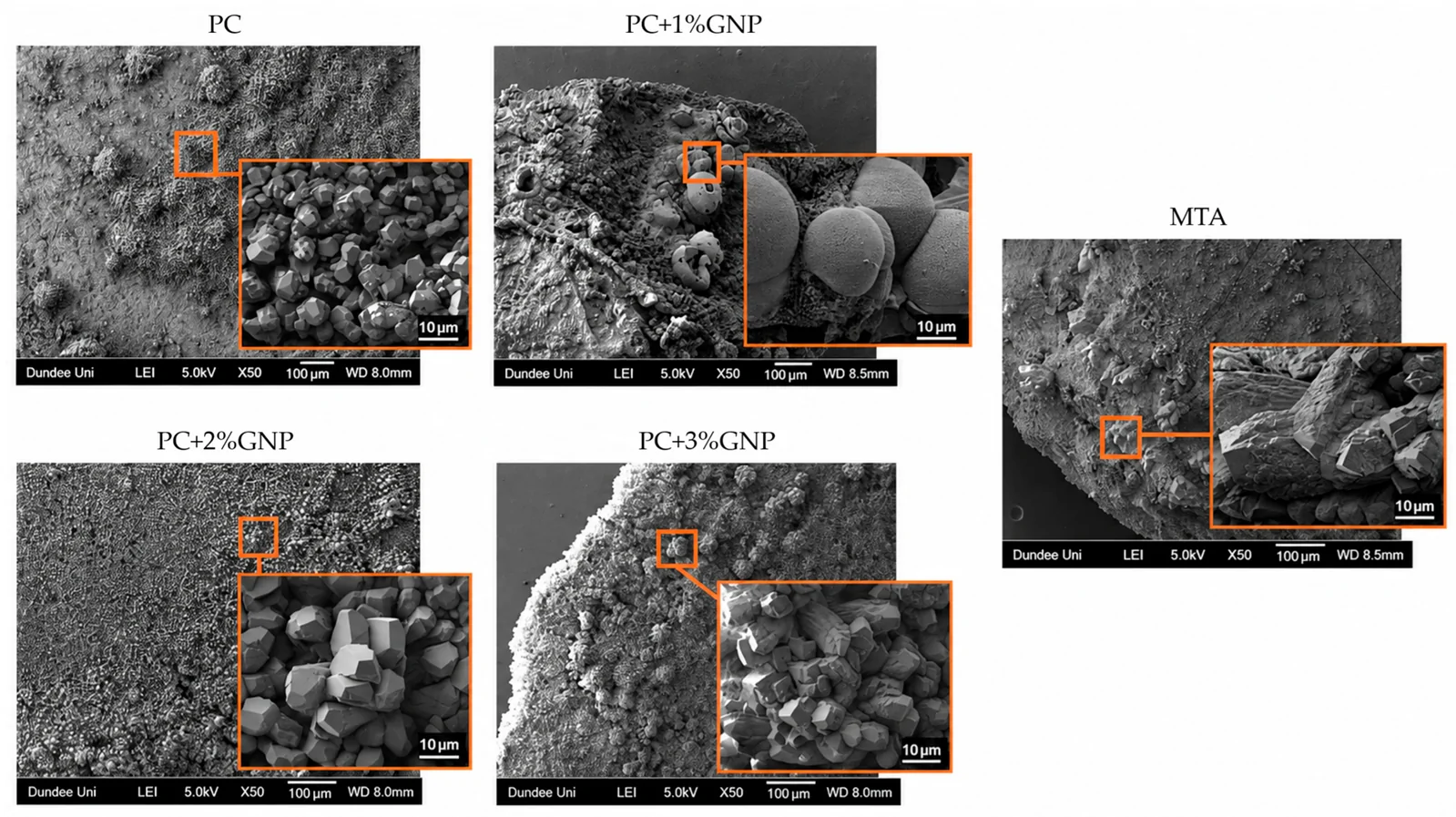
Representative scanning electron microscopy images of surface-associated deposits on unmodified Portland cement (PC), PC containing 1%, 2%, or 3% graphene nanoplatelets (GNP), and mineral trioxide aggregate (MTA) following immersion in cell culture medium at 37 °C for 14 days. Main images were acquired at ×50 magnification; scale bars = 100 µm. Boxed regions are shown in higher-magnification insets; scale bars = 10 µm. Images are presented for qualitative description of deposit morphology only. Surface coverage was not quantified, and no compositional identification was made from the SEM images. GNP, graphene nanoplatelets; MTA, mineral trioxide aggregate; PC, Portland cement.

**Figure 6:**
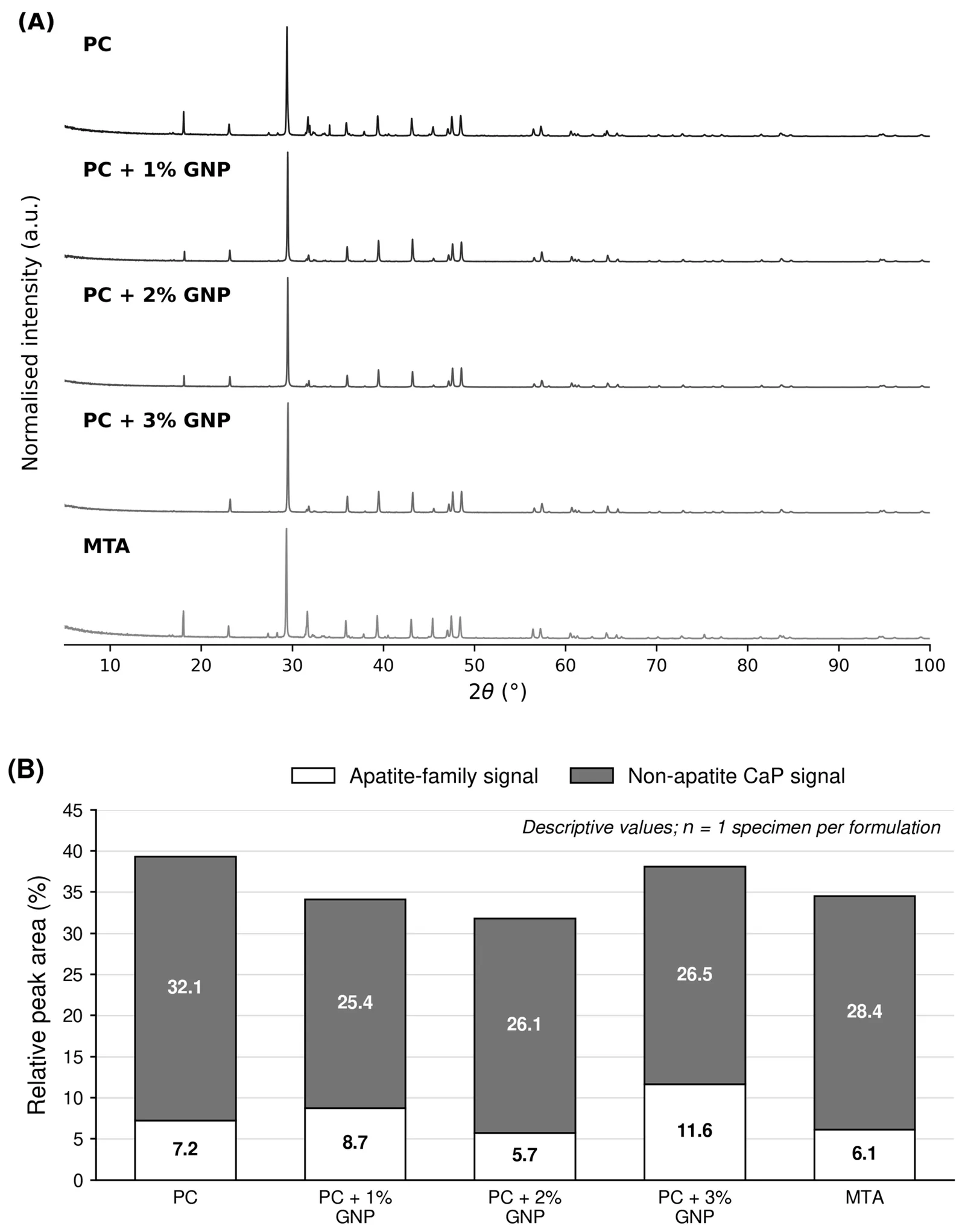
Descriptive X-ray diffraction (XRD) assessment of surface-collected material after 14 days of immersion. (**A**) Normalised and vertically offset XRD profiles of PC, PC + 1% GNP, PC + 2% GNP, PC + 3% GNP and MTA. Each profile represents one specimen per formulation and was normalised independently to its maximum intensity; vertical offsets were applied for visualisation. Corresponding unnormalised profiles and raw coordinate data are provided in Supplementary Figure S1 and Supplementary Data S1. (**B**) Descriptive relative peak-area classification of apatite-family and non-apatite calcium phosphate (CaP)-related signals. Stacked segments show the two classified components from one specimen per formulation (*n* = 1); values are displayed to one decimal place. GNP, graphene nanoplatelets; MTA, mineral trioxide aggregate; PC, Portland cement.

## Data Availability

The raw data supporting the conclusions of this article will be made available by the authors on reasonable request. Access to certain material-related data may be restricted because the tested material is the subject of a published international patent application.

## Supplementary Materials

The following supporting information can be downloaded at https://www.mdpi.com/article/10.3390/dj14100626/s1, File S1: Supplementary Figure; S1: File S2: Supplementary Data S1.

## Abbreviations

The following abbreviations are used in this manuscript:

ANOVA	Analysis of variance
CaP	Calcium phosphate
CaCl_2_	Calcium chloride
CO_2_	Carbon dioxide
DAPI	4′,6-diamidino-2-phenylindole
DMEM	Dulbecco’s Modified Eagle Medium
DMSO	Dimethyl sulfoxide
GNP	Graphene nanoplatelets
GNP-PC	Graphene nanoplatelet-modified Portland cement
HV	Vickers hardness
MEM	Minimum Essential Medium
MTA	Mineral trioxide aggregate
MTT	3-(4,5-dimethylthiazol-2-yl)-2,5-diphenyltetrazolium bromide
OD	Optical density
PBS	Phosphate-buffered saline
PC	Portland cement
PET	Polyethylene terephthalate
SEM	Scanning electron microscopy
TIV	Through-indenter viewing
VPT	Vital pulp therapy
WPS9	Human peri-radicular fibroblast cell line
XRD	X-ray diffraction
