# Supplementary material for: Graphene Nanoplatelet-Modified Portland Cement: Surface Microhardness and In Vitro Fibroblast Metabolic Activity
Source: Dent J (Basel). 2026 Oct 2;14(10):626. doi: 10.3390/dj14100626 (PMC13703387; doi:10.3390/dj14100626)
Supplement: Supplementary file 1 [file dentistry-14-00626-s001.zip › Supplementary Data S1.pdf]

Supplementary Material

Unmodified PC exhibited 7.21% apatite-family signal, 32.05% non-apatite CaP signal, and 39.26% total CaP signal. The addition of 1% GNP increased the apatite-family fraction to 8.71% while reducing the non-apatite CaP fraction to 25.43%, resulting in a total CaP signal of 34.14%. PC + 2% GNP showed 5.72% apatite-family signal, 26.13% non-apatite CaP signal, and 31.85% total CaP signal. The highest apatite-family signal was observed in the PC + 3% GNP group, reaching 11.65%, accompanied by 26.49% non-apatite CaP signal and 38.14% total CaP signal. By comparison, MTA exhibited 6.62% apatite-family signal, 28.39% non-apatite CaP signal, and 35.00% total CaP signal.

Relative to unmodified PC, PC + 3% GNP produced the greatest enhancement in apatite-family mineralisation, increasing the apatite-related signal by 4.43 percentage points (1.61-fold). PC + 1% GNP produced a smaller increase of 1.50 percentage points (1.21-fold), whereas PC + 2% GNP showed a reduction of 1.49 percentage points (0.79-fold). All GNP-containing formulations reduced the non-apatite CaP fraction by approximately 5.5–6.6 percentage points compared with PC. While total CaP signal de-creased in the PC + 1% GNP and PC + 2% GNP groups, PC + 3% GNP remained similar to unmodified PC, with only a 1.12 percentage point reduction.

To assess mineral phase redistribution, a net apatite shift index was calculated as the increase in apatite-family signal minus the decrease in non-apatite CaP signal. PC + 3% GNP exhibited the largest net apatite shift (+10.00 percentage points), followed by PC + 1% GNP (+8.13 percentage points) and PC + 2% GNP (+4.43 percentage points). These findings indicate that the 3% GNP formulation most effectively promoted conversion of the CaP-related mineral phase distribution towards apatite-family phases while pre-serving overall mineral deposition (Figure S1).

A similar trend was observed when the formulations were compared with MTA. PC + 3% GNP demonstrated the greatest apatite-family enrichment, increasing the apa-tite-related signal by 5.03 percentage points relative to MTA (1.76-fold). PC + 1% GNP also exceeded MTA, showing a 2.10 percentage point increase (1.32-fold), whereas PC + 2% GNP exhibited slightly lower apatite-family signal than MTA. All GNP-containing formulations displayed lower non-apatite CaP signal than MTA. Furthermore, total CaP signal in the PC + 3% GNP group exceeded that of MTA by 3.13 percentage points, whereas the 1% and 2% GNP groups remained
