# Supplementary material for: Graphene Nanoplatelet-Modified Portland Cement: Surface Microhardness and In Vitro Fibroblast Metabolic Activity
Source: Dent J (Basel). 2026 Oct 2;14(10):626. doi: 10.3390/dj14100626 (PMC13703387; doi:10.3390/dj14100626)
Supplement: Supplementary file 1 [file dentistry-14-00626-s001.zip › Supplementary Figure S1.pdf]

Supplementary Material

Supplementary Figure S1 presents the individual XRD profiles obtained from surface-collected material after 14 days of immersion. One specimen was examined per formulation. The corresponding descriptive relative peak-area classifications for apatite-family and non-apatite calcium phosphate-related signals are presented in Figure 6B. Because these values were derived from single specimens, they were not used to estimate quantitative phase abundance, perform statistical comparisons or infer formulation-dependent mineral conversion or enrichment. The original .xy coordinate files are provided as Supplementary Data S1

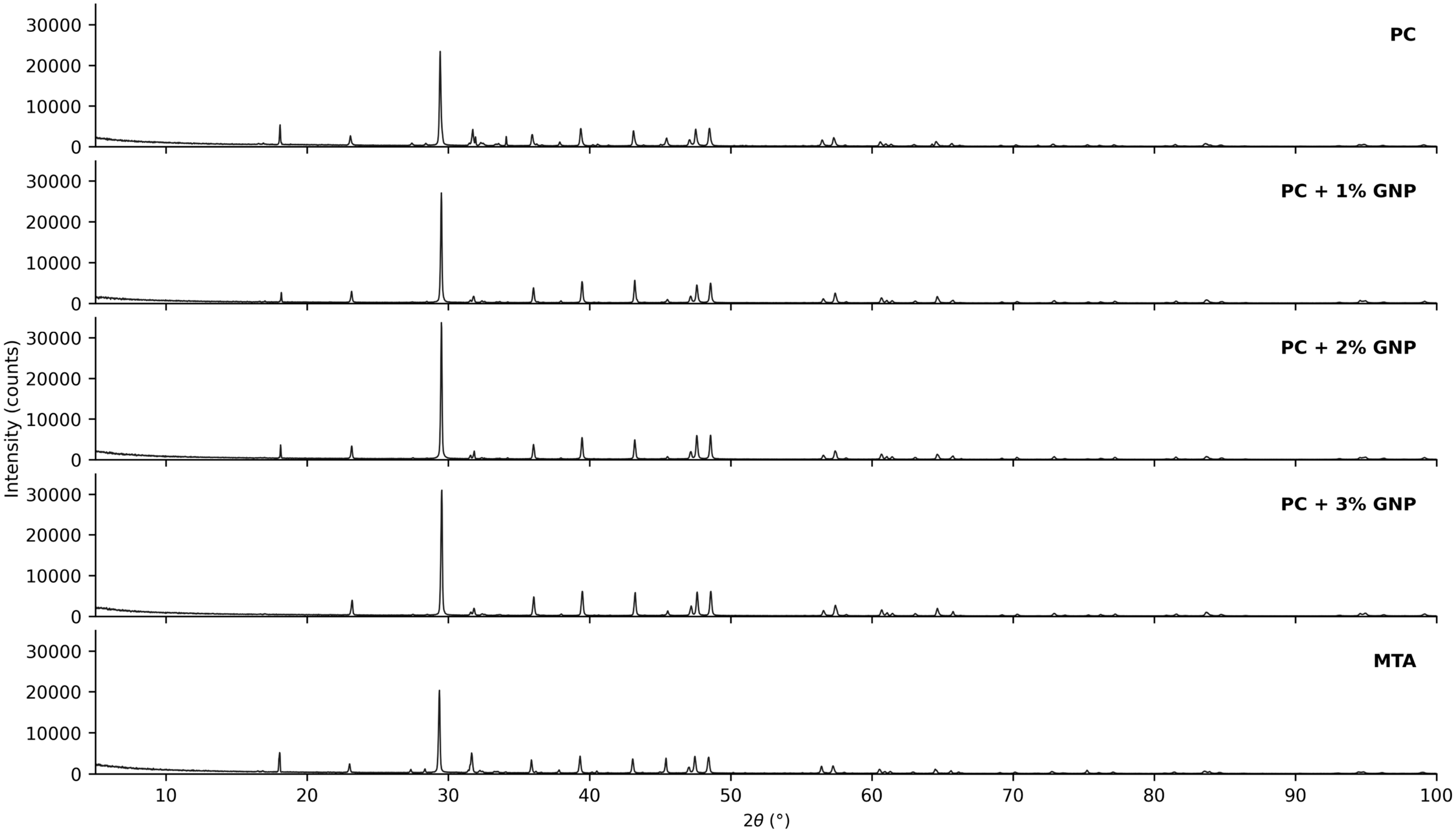

Figure S1. Individual X-ray diffraction profiles of surface-collected material from unmodified Portland cement (PC), PC containing 1%, 2% or 3% graphene nanoplatelets (GNP), and mineral trioxide aggregate (MTA) after 14 days of immersion in culture medium. One specimen was examined per formulation. Intensity values from the supplied instrument-export files are plotted against 2θ on a common intensity scale; no additional smoothing, background subtraction or phase classification was applied during preparation of this figure. The profiles are presented descriptively and were not used to estimate quantitative phase abundance or perform statistical comparisons. Surface-collected material may have included components of the underlying cement.
